# The Role of Intranasal and Inhaled Therapies in the Management of Cluster Headache: A Network Meta-analysis

**DOI:** 10.1055/s-0046-1827860

**Published:** 2026-10-23

**Authors:** Talal Aljeraisi

**Affiliations:** 1 Department of Otolaryngology–Head and Neck Surgery, Faculty of Medicine, Taibah University, Al-Madinah Al-Munawwarah, Saudi Arabia

**Keywords:** cluster headache, episodic migraine, nasal spray, zolmitriptan, sumatriptan, capsaicin

## Abstract

**Introduction:**

There are limited evidence-based nasal therapies available to treat cluster headaches. Likewise, there are not many head-to-head treatment comparisons available because most clinical trials have only compared therapies versus placebos.

**Objective:**

To compare different nasal therapies for cluster headache.

**Data Synthesis:**

We used four databases to search for records and then screened them for eligibility. In randomized studies, the risk of bias was assessed using the Cochrane tool for randomized clinical trials. The data were then retrieved and used in double-arm analyses using the R Software (free and open source). Zolmitriptan 10 mg and oxygen demonstrated the greatest efficacy for headache relief, while oxygen ranked highest for pain-free outcomes. Zolmitriptan 10 mg was most effective in reducing rescue medication use. Across outcomes, zolmitriptan 10 mg and oxygen consistently ranked as the most effective treatments, with no evidence of inconsistency.

**Conclusion:**

Intranasal therapeutic sprays expand the treatment options and may provide better results, more rapid relief, and higher patient satisfaction than oral or injectable medications. Although many intranasal therapeutic sprays showed variable efficacy in cluster headache relief, more randomized controlled trials on a large sample of patients with a longer follow-up duration are needed.

## Introduction

A main headache disease, cluster headache (CH) affects between 0.5 to 1.0 per 1 thousand individuals. 1 Severe, unilateral discomfort around the eyes is the hallmark of these episodes, which are also accompanied by particular autonomic symptoms on the same side, such as weeping, redness, congestion, runny nose, drooping eyelid, and a restless or agitated mood. 2 Most victims of the disorder experience CHs that continue for weeks to months, interspersed with intervals of remissions. 3 The condition usually follows a pattern of recurrent episodes. Ten to 15% of patients experience persistent CHs, which are characterized by recurrent attacks lasting up to 3 hours and happening up to 8 times a day. 2 Due to higher healthcare expenses and longer sick leave durations, this ailment has a substantial negative impact on both individuals and society as a whole. 1 Notwithstanding the severity, it is still unknown what fundamental processes set off cluster periods and isolated attacks, which leaves little in the way of precise preventative drugs and therapy choices 3 4

When someone has episodic cluster headache (ECH), they get headaches on less than 15 days per month. On the other hand, headaches for at least 15 days a month for 3 months or longer, with at least 8 of those days exhibiting migraine symptoms, are considered chronic cluster headache (CCH). 2 This distinction is important since CCH is associated with far greater difficulties on a personal and societal level, frequently coexists with more comorbid diseases, and may be associated with long-lasting and potentially increasing brain abnormalities. 5 6

Intranasal therapies have emerged as a potential non-invasive option for the acute management of CH, particularly in situations in which rapid onset of action is required. The intranasal route offers several theoretical advantages, including avoidance of gastrointestinal absorption, faster systemic delivery, and ease of administration during severe attacks when oral intake may be impractical. These features are especially relevant in CH, which is characterized by abrupt onset and intense pain requiring rapid relief. Despite these advantages, evidence supporting intranasal treatments in CH remains limited and heterogeneous. While intranasal triptans, such as zolmitriptan and sumatriptan, have demonstrated efficacy in randomized trials, their onset of action is generally slower compared with subcutaneous formulations, which remain the standard of care for acute attacks. Additionally, variability in nasal absorption, device-dependent delivery, and inconsistent patient response may limit their effectiveness in clinical practice. Overall, intranasal therapies may serve as an alternative option for selected patients; however, current evidence does not support their superiority over established treatments, and further high-quality comparative studies are needed to better define their role in CH management. 7

There are not many evidence-based nasal treatments available for treating CHs, neither are there many direct comparisons between them. The present study used available data and subgroup analysis, when feasible, to assess and compare the benefits and drawbacks of various nasal therapies for CH. We combined direct and indirect evidence from the body of literature already in existence by performing a network meta-analysis. Our objective was to close the knowledge gap by providing doctors with comparative data on acute therapies for CH.

## Methods

The present investigation was conducted in exact accordance with the protocols described in the Cochrane Handbook for Systematic Reviews of Interventions 8 and the Preferred Reporting Items for Systematic Reviews and Meta-Analyses (PRISMA). 9 The Grading of Recommendations Assessment, Development, and Evaluation 10 was used to evaluate the study's quality.

### Inclusion and Exclusion Criteria

Studies that included individuals with CHs and did not place limitations on participant age or kind of nasal therapy were deemed appropriate for inclusion. Research that was conducted in vitro, concentrated on non-nasal treatments for headaches, had duplicate datasets, reviews, book chapters, dissertations, editorials, letters to the editor, publications that were only available in abstract form, conference abstracts, articles written in non-English, case studies, or cross-sectional analyses were all excluded.

### Study Eligibility Criteria

Eligible studies included randomized controlled trials (RCTs) as well as prospective and open-label studies evaluating intranasal therapies for headache relief. No restriction was placed on blinding status. Uncontrolled studies were included if they reported relevant outcomes. The inclusion of non-randomized designs was intended to maximize available evidence but was considered during interpretation of results.

### Literature Search

The Cochrane Central Register of Controlled Trials (CENTRAL), Web of Science (WOS), Scopus, and MEDLINE databases were thoroughly searched from the time each database was created until March 1, 2024. Using the following search terms: (( *Cluster headache* ) AND ( *Sumatriptan* OR *Lidocaine* OR *Dihydroergotamine* OR *Ketamine* OR *Oxymetazoline* OR *Zolmitriptan* OR *Ergotamine* OR *Bupivacaine* OR *Capsaicin* OR *Oxytocin* OR *Melatonin* OR *Calcitonin gene-related peptide* OR (CGRP) OR *Dexamethasone* OR *Propofol* OR *Magnesium sulfate* OR “ *intranasal medication* OR *nasal administration* OR *nasal spray therapy* OR *nasal* *delivery* )). Novel therapies were included without limitations to provide thorough coverage. In addition, a manual examination of all reviews that were accessible about the management of cluster headache was conducted to find any possible further research. No language constraints were used when doing the search.

### Data Extraction

Extracted data included the exclusion and inclusion criteria, study design, baseline demographics of the patients involved in our study, and safety and efficacy outcomes.

### Outcomes

The outcomes considered in the current study were efficacy, headache relief, pain-free state, and use of rescue medications. Safety outcomes included any adverse events.

### Risk of Bias Assessment

We used the Cochrane risk of bias tool (ROB 2) in accordance with the recommendations given in the Cochrane handbook to assess the possibility of bias. 8 The following domains were evaluated: (1) bias arising from the randomization process, (2) bias due to deviations from intended interventions, (3) bias due to missing outcome data, (4) bias in measurement of the outcome, and (5) bias in selection of the reported result.

### Network Geometry and Assumptions

The geometry of the treatment network was explored by constructing network plots for each outcome, in which nodes represent interventions and edges represent direct comparisons between treatments. The size of nodes reflects the number of participants, and the thickness of edges reflects the number of studies contributing to each comparison.

The assumption of transitivity was assessed by evaluating the similarity of study and patient characteristics across treatment comparisons, including baseline demographics, disease severity, and study design.

#### Certainty of Evidence Assessment (GRADE)

The certainty of evidence was assessed using the Grading of Recommendations Assessment, Development and Evaluation (GRADE) approach. The following domains were evaluated: risk of bias, inconsistency, indirectness, imprecision, and publication bias, in accordance with the recommendations of the Cochrane Handbook.

### Statistical Analysis

We used the meta and netmeta packages in the R Software (free and open source)to do network analysis on the data to evaluate and combine it. We used the risk ratio (RR) in conjunction with a 95%CIs for binary outcomes. Heterogeneity was assessed at both the pairwise and network levels. For direct pairwise comparisons, statistical heterogeneity was quantified using the I ^2^ statistic and τ ^2^ . Within the network meta-analysis, heterogeneity was evaluated using the between-study variance (τ ^2^ ) estimated from the network model rather than relying solely on a single overall I ^2^ value. Because heterogeneity may differ across comparisons, I ^2^ values from pairwise analyses were interpreted per direct comparison when sufficient studies were available, whereas τ ^2^ was used as the principal measure of global between-study heterogeneity in the network. Treatments were ranked using *p* -scores, which estimate the mean probability that a treatment is among the most effective. Global inconsistency was assessed using the design-by-treatment interaction model.

## Results

### Data Collection and Characteristics of the Studies Included

Database searches identified a total of 1,882 articles: 538 from PubMed, 414 from WOS, 789 from Scopus, and 141 from Cochrane. We screened the articles and removed duplicates. A total of 1,057 records were eligible for title and abstract screening. We excluded 985 records using title and abstract, and the remaining 72 articles' full texts were then screened to identify research that qualified. Finally, a total of 13 records 11 12 13 14 15 16 17 18 19 20 21 22 23 were included in the qualitative synthesis, and 7 records 12 14 17 20 21 22 23 of them were included in the quantitative synthesis as shown in the Preferred Reporting Items for Systematic Reviews and Meta-Analyses (PRISMA) flow diagram ( Fig. 1 ).

**Fig. 1:**
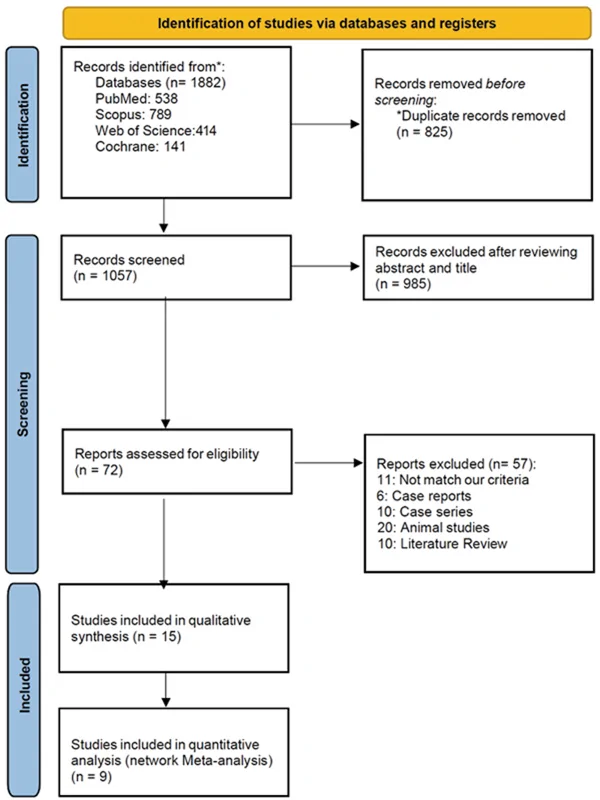
Preferred Reporting Items for Systematic Reviews and Meta-Analyses (PRISMA) flow diagram.

The sample sizes of the RCTs included ranged from 12 to 118 patients. Different doses of nasal therapies were compared with others or to placebo. The included studies' follow-up times ranged from 15 minutes to 15 days. The average ages of the patients included ranged from 19 to 68 years. Males ranged in representation from 3 to 97 in each record. A list of the baseline characteristics of included patients is provided in Table 1 , and Table 2 lists the summary of the included studies.

**Table 1:** Baseline characteristics of the patients included in the study

Study ID	Study arms, (n)	Sample size	Study design	Follow-up	Age (year)	Gender (men/women)	Treatment
Andersson et al.,1986 19	DHE nasal spray or placebo	25	Double-blind trial	NR	40 (range, 26–60)	20/5	Dihydroergotamine nasal spray
Rapoport et al.,2007 22	Zolmitriptan 5 mg, zolmitriptan 10 mg, and placebo	78	Double-blind, randomized	60 minutes	45.2 ± 11.2	31/14	Zolmitriptan nasal spray (ZNS)
Cho et al.,2024 20	Zolmitriptan-oxygen	40	Randomized, crossover, multicenter study	120 minutes	40.0 (IQR, 31.0–44.8 years),	28 (87.5) males	Home oxygen concentrators
Cittadini et al.,2006 17	placebo, 5 mg of zolmitriptan nasal spray, 10 mg of zolmitriptan	92	A randomized, placebo-controlled, double-blind crossover study	30 minutes	40 ± 10	80/12	Intranasal zolmitriptan
Cittadini et al.,200821	placebo, zolmitriptan nasal spray 5 and 10 mg	92	Randomized placebo-controlled double-blind crossover study	30 minutes	40 ± 10	80/12	Zolmitriptan nasal spray (ZNS)
Costa et al.,2000 18	Cocaine hydrochloride, lidocaine, saline	15	double-blind placebo-controlled	45 minutes	ECH:36.8 ± 5.6, CCH:37.8 ± 10.4	13/2	10% solution of cocaine hydrochloride (1 ml, mean amount per application 40 ± 50 mg), or 10% lidocaine (1 ml), or saline
Hardebo and Dahlöf,1998 11	Sumatriptan nasal spray, sumatriptan subcutaneous injection	25	open, randomized	15 minutes	26 to 68	22/3	Sumatriptan nasal spray 20 mg and sumatriptan subcutaneous injection 6 mg
Marks et al.,1993 14	Capsaicin, placebo	17	Double-blind placebo-controlled trial	15 days	NR	3/10 women	3% camphor in 0.025% capsaicin cream and placebo (3% camphor in vehicle cream).
Nilsson et al.,2002 15	HBO, sham	16	Double blind placebo-controlled crossover study	one week	20–62	12/4 women	HBO (100% oxygen, hyperbaric chamber, 250 kPa for 70 minute) and SHAM (10% oxygen in nitrogen, hyperbaric chamber, 250 kPa for 70 minute)
Petersen et al.,2022 16	Intranasal ketamine	23	open-label trial	30 minutes	51 (27–60)	16/7	15 mg of intranasal ketamine every 6 minutes
Robbins,1995 12	Intranasal lidocaine	30	Prospective study	15 minutes	23 and 63	13 males	4% lidocaine solution to use intranasally as an abortive therapy
Schuh-Hofer et al.,2002 13	Sumatriptan nasal spray	12	open-label study	15 minutes	25–60	12 males	20 mg sumatriptan nasal spray
Van Vliet et al.,2003 23	Sumatriptan nasal spray, placebo.	118	Randomized placebo-controlled double-blind study	45 minutes	43 ± 11	97/21	20 mg sumatriptan nasal spray

**Abbreviations** : CCH, chronic cluster headache; CH, cluster headache; DHE, dihydroergotamine; ECH, Episodic cluster headache; HBO, oxygen, hyperbaric chamber; NR, not reported; ZNS, zolmitriptan nasal spray.

**Table 2:** Summary of the studies included in the study

Study ID	ECH/CCH	Inclusion/exclusion criteria	Conclusion
Andersson et al.,1986 19	ECH	NR	The nasal administration of 1 mg of DHE corresponds to 10 mg given orally, and the relative bioavailability of DHE nasal spray is 40% of that for intramuscular DHE. These findings are of importance in the treatment of patients with acute vasomotor headache.
Rapoport et al.,2007 22	37 patients had ECH and 15 had CCH.	The included subjects aged 18–65 years, with a diagnosis of ECH or CCH, according to the second edition of the International Classification of Headache Disorders.	Both doses had higher pain-free rates than placebo at 30 minutes (placebo: 20%, ZNS: 5 mg 38.5%, ZNS: 10 mg 46.9%). Side effects were mild and seen in 16% of those attacks treated with placebo, 25% of attacks treated with ZNS 5 mg, and 32.7% treated with ZNS 10 mg.
Cho et al.,2024 20	ECH	This prospective study included adults aged 18–65 years with ECH from headache clinics at eight hospitals in South Korea between November 2021 and November 2022.	Oxygen treatment administered using two home oxygen concentrators resulted in better pain relief than oral Zolmitriptan in patients with ECH. The results suggested that home oxygen concentrators are capable of efficiently supplying oxygen in a similar manner to using an oxygen tank.
Cittadini et al.,2006 17	59 patients ECH and 33 CCH.	The excluded patients were unsuitable for zolmitriptan tablet or nasal spray use in the country in which the study was being conducted according to the drug label or regulatory approved use in that country. Also, patients with more than 2 risk factors for cardiovascular disease, patients using regular ergotamine derivatives or analgesics, and patients with an ear, nose, and throat disorder that would preclude the use of intranasal zolmitriptan were excluded.	Zolmitriptan in doses of 5 mg, and 10 mg intranasal spray is effective within 30 minutes and well tolerated in the treatment of acute cluster headache.
Cittadini et al.,2008 21	40 patients had ECH and 29 had CCH	Excluded patients were unsuitable for use of zolmitriptan tablet or nasal spray in the country where the study was performed according to the drug label or regulatory approved use in that country. Also, patients with more than two risk factors for cardiovascular disease, patients using regular ergotamine derivatives or analgesic, and patients with an ear, nose and throat disorder that would preclude the use of intranasal zolmitriptan were excluded.	Zolmitriptan 5 and 10 mg intranasal is effective within 30 minutes and well tolerated in the treatment of acute cluster headache.
Costa et al.,2000 18	6 patients had ECH, 9 patients had CCH	NR	The findings indicated that the local administration of the anesthetic agents cocaine and lidocaine is effective on NTG-induced CH attacks, and may be used in the symptomatic treatment of this disorder.
Hardebo and Dahlöf,1998 11	3 CCH, 22 ECH	NR	As an overall judgement, only 2 out of all patients preferred nasal spray to injection. Sumatriptan nasal spray 20 mg/dose is less effective than subcutaneous injection in relieving pain in the great majority of cluster headache sufferers.
Marks et al.,1993 14	5 patients with ECH, and 8 patients with CCH	Patients who met the inclusion criteria were seen at the New England Center for Headache (Stamford, CT) and randomized to receive either capsaicin or placebo. The study drug was applied, via cotton-tipped applicator, one-half inch up the nostril, ipsilateral to the side of the head on which the patient experienced the headaches. The drug was applied in this manner twice daily for 7 days.	ECH patients appeared to benefit more than CCH patients. These results indicated that intranasal Capsaicin may provide a new therapeutic option for the treatment of this disease.
Nilsson et al.,2002 15	12 patients with ECH, and 4 patients with CCH	They followed the International Headache Society criteria, and patients were excluded if they had used any CH prophylactic treatment during the present period, had heart or circulatory disorders, present/history of ear disorders or were pregnant or nursing.	HBO seems to be effective in reducing headache severity, at least in patients with ECH, although a powerful placebo response cannot be ruled out.
Petersen et al.,2022 16	CCH	NR	Intranasal ketamine may be an effective acute treatment for CH at 30 minutes but should be tested in a larger controlled design. Patients and physicians should be conscious of the abuse potential of ketamine.
Robbins,1995 12	ECH	NR	In this study, intranasal lidocaine was only a marginally helpful therapy for CH. However, because of the ease of administration and lack of side effects, lidocaine may remain worthwhile as an adjunctive medication.
Schuh-Hofer et al.,2002 13	4 patients with CCH, 6 patients with ECH	They followed the International Headache Society criteria. Other exclusion criteria were present or previous relevant neurological or psychiatric illnesses, relevant elevation of liver enzymes, electrocardiogram abnormalities, pregnancy or lactation, women without adequate contraceptive precautions or patients who were on monoamine oxidase inhibitors.	20 mg Sumatriptan nasal spray might be an alternative therapy for the treatment of CH attacks, but double-blind studies are needed to further evaluate its efficacy.
Van Vliet et al.,2003 23	89 patients with ECH, 29 patients with CCH	Only patients with the symptom at baseline were included.	Sumatriptan nasal spray was effective and well tolerated in the acute treatment of cluster headache attacks of at least 45 minutes duration.

**Abbreviations** : CCH, chronic cluster headache; CH, cluster headache; DHE, dihydroergotamine; ECH, episodic cluster headache; HBO, oxygen, hyperbaric chamber; NR, not reported; NTG, nitroglycerin-induced attacks; ZNS, zolmitriptan nasal spray.

### Risk of Bias (ROB) Assessment

The risk of bias assessment demonstrated substantial variability in methodological quality across the included studies. Five studies were rated as having some concerns, primarily due to issues related to missing outcome data, deviations from intended interventions, and insufficient reporting. The remaining seven studies were classified as having high risk of bias, largely driven by limitations in randomization processes, lack of blinding, and potential bias in outcome measurement. At the domain level, high risk of bias was most frequently observed in the domains of deviations from intended interventions and outcome measurement, reflecting limited blinding and potential performance and detection bias in several studies. Missing outcome data was commonly rated as “some concerns” across most studies, indicating incomplete reporting or unclear handling of attrition. In contrast, studies with selective reporting were generally assessed as having low risk of bias or some concerns, with no studies clearly demonstrating high risk in this domain. Figs. 2A,B describe the quality assessment of the included articles.

**Fig. 2:**
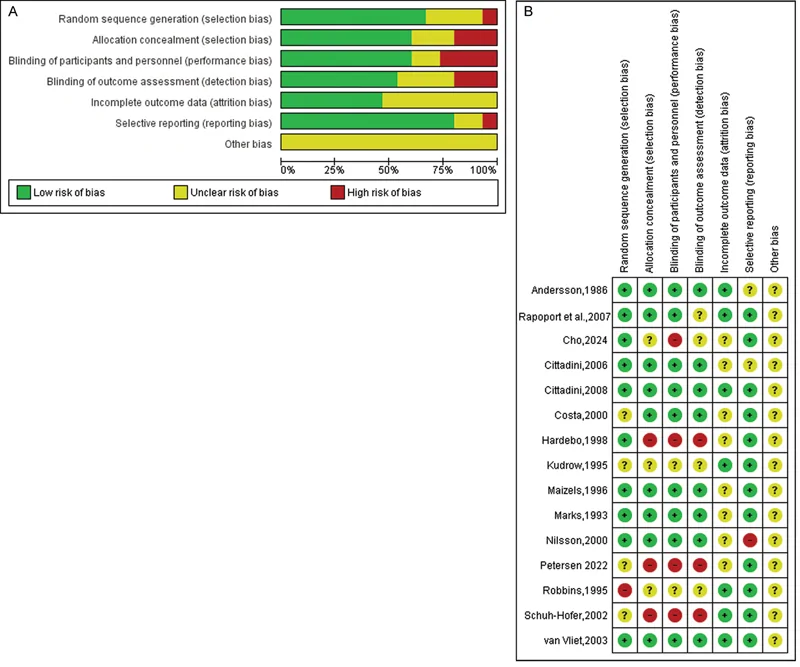
( **A,B** ) and 2B Quality assessment of the articles included.

### Headache Relief Rates at 15 or 30 minutes

Compared with placebo, all active treatments demonstrated statistically significant improvement in headache relief. Zolmitriptan 10 mg showed the greatest effect (relative risk [RR] = 2.52, 95%CI: 1.93–3.30), followed by oxygen (RR = 2.48; 95% I: 1.53–4.01). Zolmitriptan 5 mg was also effective compared with placebo, although with a smaller magnitude (RR = 1.68; 95%CI: 1.25–2.26). Zolmitriptan 10 mg ranked highest ( *p* -score = 0.84), followed by oxygen (0.82), zolmitriptan 5 mg (0.34), and placebo (0.00). These results indicate that zolmitriptan 10 mg and oxygen are the most effective treatments for headache relief. The results showed no evidence of inconsistency (Q = 2.44; *p* = 0.66). The between-design inconsistency component was negligible, indicating strong agreement between direct and indirect evidence. League table comparisons showed that all active treatments were more effective than placebo, with oxygen (RR = 2.48; 95%CI: 1.53–4.01), zolmitriptan 10 mg (RR = 2.50; 95%CI: 1.92–3.33), and zolmitriptan 5 mg (RR = 1.67; 95%CI: 1.25–2.22) demonstrating significant benefits, as shown in Fig. 3 .

**Fig. 3:**
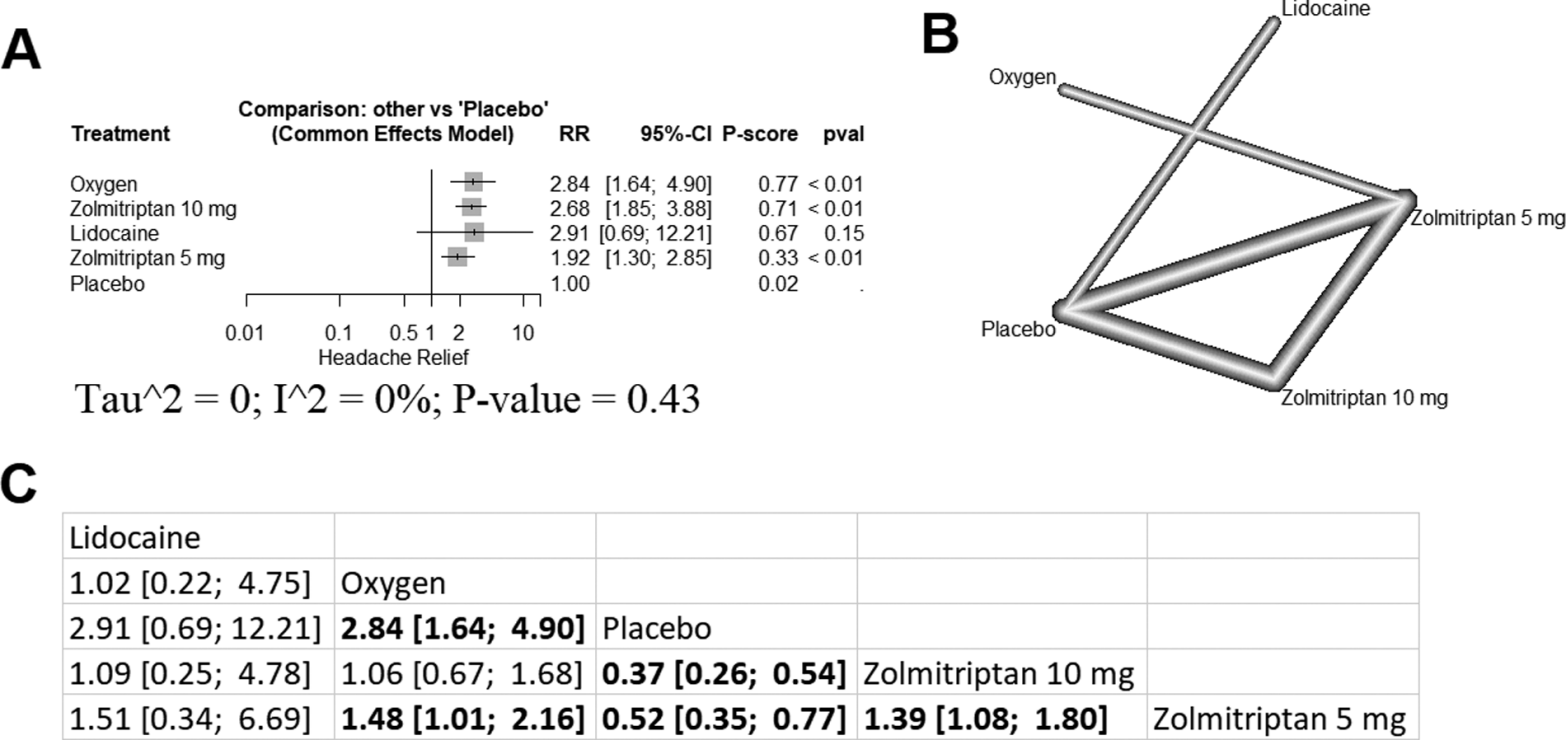
Headache relief. ( **A** ) Forest plot showing pairwise and network effect estimates expressed as risk ratio with 95%CIs. ( **B** ). Network map illustrating the geometry of direct treatment comparisons included in the network. Nodes represent different interventions, with node size proportional to the total sample size, and edges represent available direct evidence, with line thickness reflecting the number of contributing studies. Indirect comparisons were derived statistically through connected treatment pathways and are not displayed as separate edges in the network map. ( **C** ). League table (pairwise comparisons) ( **D** ). Surface under the cumulative ranking ordination diagram displaying the cumulative ranking probabilities of each intervention.

### Pain Free Rates

Compared with placebo, all active treatments demonstrated improved rates of achieving a pain-free state, with varying magnitudes of effect. Zolmitriptan 10 mg (RR = 3.08; 95%CI: 2.11–4.48), oxygen (RR = 4.18; 95%CI: 1.85–9.44), and zolmitriptan 5 mg (RR = 1.91; 95%CI: 1.27–2.88) showed statistically significant benefits. Capsaicin demonstrated a large point estimate (RR = 4.44), although with wide confidence intervals crossing unity (95%CI: 0.65–30.44), indicating substantial uncertainty. Oxygen ranked highest ( *p* -score = 0.81), followed by capsaicin (0.73) and zolmitriptan 10 mg (0.64). Zolmitriptan 5 mg (0.30) and placebo (0.02) ranked lower. These findings suggest that oxygen and capsaicin have the highest probability of being among the most effective treatments for achieving pain-free outcomes, although the estimate for capsaicin should be interpreted with caution due to imprecision. inconsistency testing using the design-by-treatment interaction model did not detect significant inconsistency within the network (Q = 1.29; df = 4; *p* = 0.86), indicating good agreement between direct and indirect evidence. League table comparisons indicated that several active treatments were more effective than placebo in achieving pain-free outcomes. ( Fig. 4 ).

**Fig. 4:**
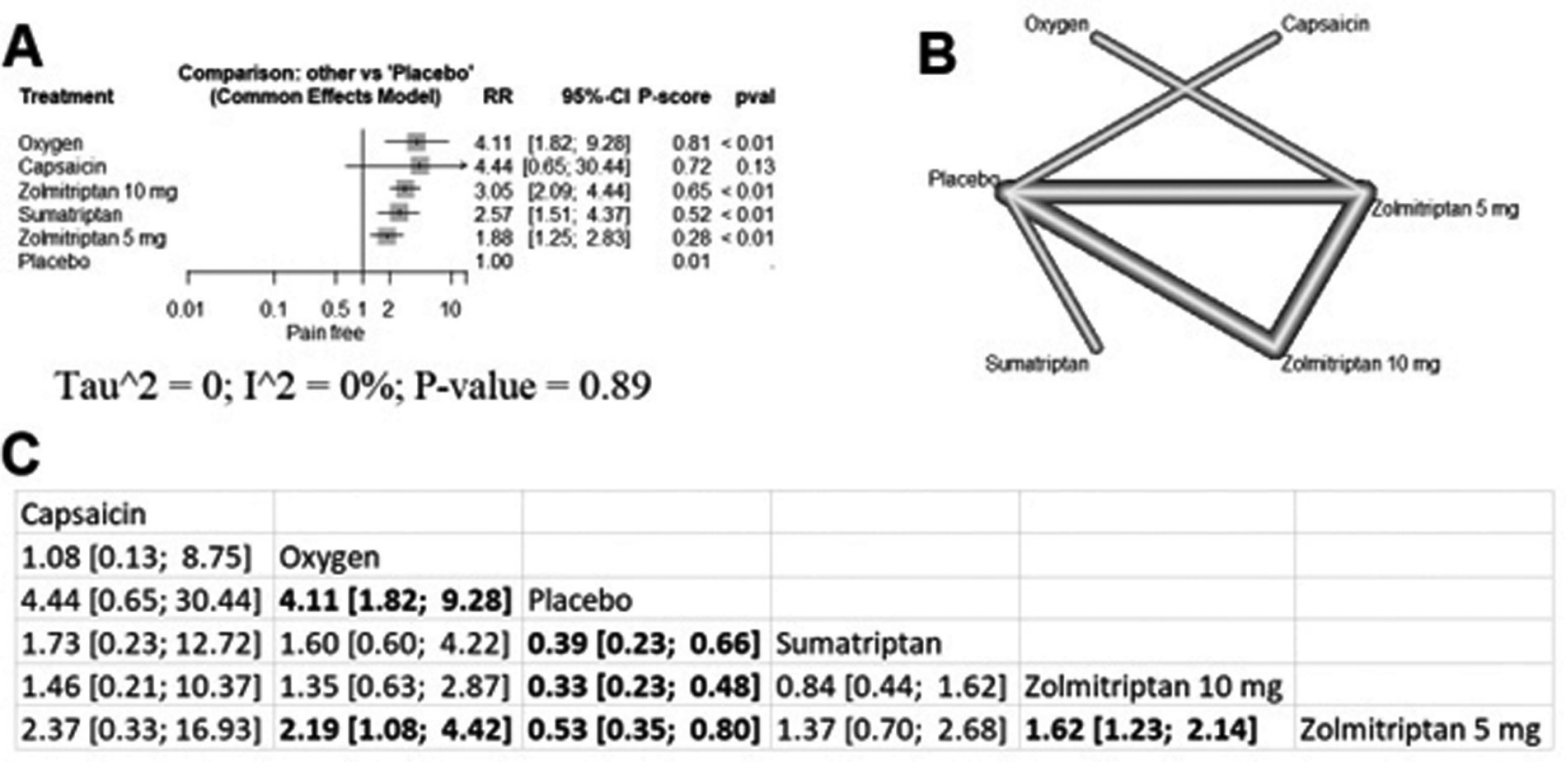
Pain free. ( **A** ). Forest plot showing pairwise and network effect estimates expressed as risk ratio with 95%CIs. ( **B** ). Network map illustrating the geometry of direct treatment comparisons included in the network. Nodes represent different interventions, with node size proportional to the total sample size, and edges represent available direct evidence, with line thickness reflecting the number of contributing studies. Indirect comparisons were derived statistically through connected treatment pathways and are not displayed as separate edges in the network map. ( **C** ) League table (pairwise comparisons). ( **D** ). Surface under the cumulative ranking ordination diagram displaying the cumulative ranking probabilities of each intervention.

### Use of Rescue Medications

Compared with placebo, zolmitriptan 10 mg was associated with a statistically significant reduction in the use of rescue medications (RR = 0.64; 95%CI: 0.46–0.89). Zolmitriptan 5 mg demonstrated a borderline reduction compared with placebo (RR = 0.74; 95%CI: 0.55–1.00), while oxygen did not show a statistically significant difference (RR = 0.57; 95%CI: 0.26–1.24). Zolmitriptan 10 mg ranked highest ( *p* -score = 0.84), followed by oxygen (0.82), zolmitriptan 5 mg (0.34), and placebo ( *p* -score < 0.001). These findings suggest that zolmitriptan 10 mg has the greatest likelihood of reducing the need for rescue medications. Inconsistency within the network (Q = 0.76; *p* = 0.68), indicated agreement between direct and indirect comparisons. League table comparisons showed that zolmitriptan 10 mg significantly reduced the use of rescue medications compared with placebo ( Fig. 5 ).

**Fig. 5:**
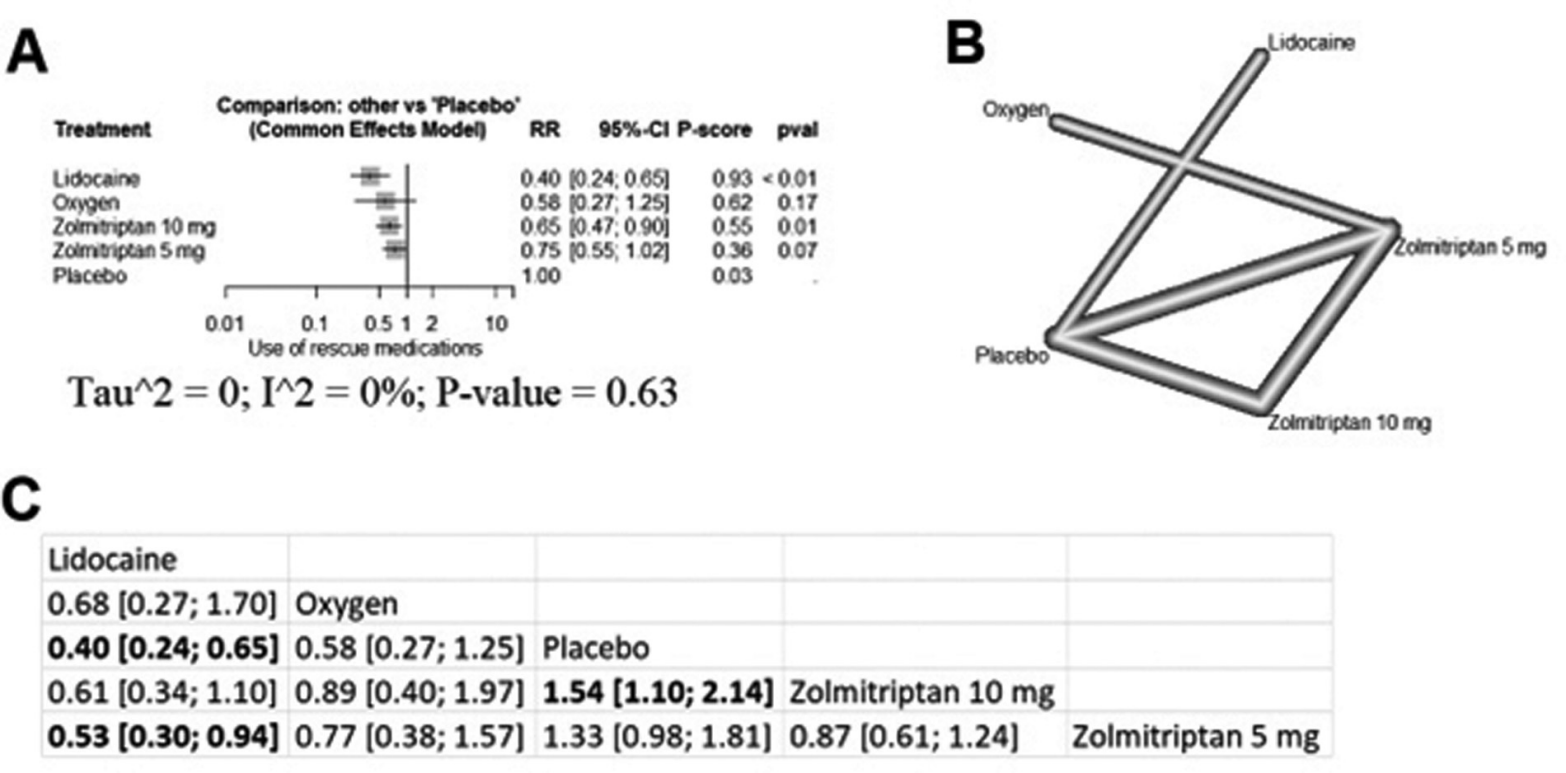
Use of rescue medications. ( **A** ). Forest plot showing pairwise and network effect estimates expressed as risk ratio with 95%CIs. ( **B** ). Network map illustrating the geometry of direct treatment comparisons included in the network. Nodes represent different interventions, with node size proportional to the total sample size, and edges represent available direct evidence, with line thickness reflecting the number of contributing studies. Indirect comparisons were derived statistically through connected treatment pathways and are not displayed as separate edges in the network map. ( **C** ). League table (pairwise comparisons) ( **D** ). Surface under the cumulative ranking ordination diagram displaying the cumulative ranking probabilities of each intervention.

#### Certainty of Evidence (GRADE assessment)

The certainty of evidence was primarily downgraded due to methodological limitations across the studies included, particularly risks of bias related to randomization and blinding, despite low statistical heterogeneity across analyses.

## Discussion

Improved understanding of the pathophysiology of CH has led to the identification of new potential therapeutic targets. Although conventional treatments, such as oxygen therapy, triptans, and calcium channel blockers, remain the foundation of management, their effectiveness is not consistent across all patients, and some individuals may experience limited benefit or adverse effects. Therefore, there is increasing interest in investigating alternative and adjunctive approaches to enhance treatment outcomes in this difficult-to-manage condition. 7

Based on our analysis, we found that all active treatments demonstrated superiority over placebo. For headache relief at 15 to 30 minutes, zolmitriptan 10 mg showed the greatest effect (RR = 2.52), followed closely by oxygen (RR = 2.48), with zolmitriptan 5 mg showing a smaller but significant benefit; ranking favored zolmitriptan 10 mg ( *p* -score = 0.84) and oxygen (0.82), with no evidence of inconsistency ( *p* = 0.66). For pain-free rates, oxygen ranked highest ( *p* -score = 0.81), followed by capsaicin (0.73), and zolmitriptan 10 mg (0.64); zolmitriptan 10 mg, oxygen, and zolmitriptan 5 mg demonstrated significant effects, while capsaicin showed high uncertainty due to wide confidence intervals, with no detected inconsistency ( *p* = 0.86). Regarding rescue medication use, zolmitriptan 10 mg significantly reduced the need for rescue therapy (RR = 0.64) and ranked highest ( *p* -score = 0.84), whereas zolmitriptan 5 mg showed borderline benefit, and oxygen was not statistically significant; again, no inconsistency was observed ( *p* = 0.68). Overall, zolmitriptan 10 mg and oxygen consistently emerged as the most effective treatments across outcomes, with robust and internally consistent network estimates.

The main initial treatment for CHs is oxygen therapy; however, its usage may be restricted due to issues with insurance approval and maintaining oxygen tanks. Oxygen concentrators could be a good substitute for conventional tank-based oxygen therapy since they produce oxygen-enriched gas by removing nitrogen from the ambient air. 20 This is consistent with our findings, which showed that oxygen was superior to placebo in increasing headache relief rates at 15 to 30 minutes. Similarly, we found that Zolmitriptan 10 and 5 mg had higher relief rates than placebo, which is in agreement with the results reported by Rapoport et al. 22

Also, Bahra et al. 24 reported that zolmitriptan 5 mg showed substantial superiority over placebo in 3 out of 4 secondary measures, and zolmitriptan 10 mg significantly outperformed placebo across all other secondary measures in individuals with ECHs. On the other hand, among patients with persistent CHs, there was no discernible difference in the efficacy of zolmitriptan (either 5 mg or 10 mg) when compared with placebo at any assessed endpoint. It was discovered that zolmitriptan at doses of 5 mg and 10 mg was well tolerated.

### Limitations and Strengths

We provide a low-quality rating to all the outcomes' evidence. Most data come from research with ambiguous or minimal bias risks. The outdated publication dates of several studies allow us to rationalize the ambiguous factors. Additionally, although RCTs were prioritized, we also included prospective and open-label studies that met our predefined eligibility criteria, to capture the available evidence comprehensively. Study selection and reporting adhered to the PRISMA checklist, and methodological conduct followed the recommendations of the Cochrane Handbook. This is the first network meta-analysis that contrasts several intranasal therapies with a placebo, as per our research. The combined data were all identical. We observed, however, certain restrictions: (1) The relatively short follow-up period; (2) The modest number of included studies with small sample sizes; and (3) Some studies' use of distinct pain scales. Although statistical heterogeneity appeared low in the available analyses, the small number of studies per comparison may have limited the ability of I ^2^ to detect true between-study heterogeneity. Finally, the inclusion of non-randomized and open-label studies may introduce potential bias and limit the internal validity of the findings. Future analyses restricted to RCTs are warranted to confirm the robustness of these results.

## Conclusion

Oxygen and triptans (zolmitriptan and sumatriptan) were associated with improved headache relief, higher pain-free rates, and reduced need for rescue medications compared with placebo. Zolmitriptan 10 mg and oxygen demonstrated the most consistent and robust effects across outcomes. In contrast, the evidence for capsaicin and lidocaine remains uncertain due to imprecision and limited data. Overall, the certainty of evidence is moderate to low, and these findings should be interpreted with caution.
